# Hypoallergenic Variant of the Major Egg White Allergen Gal d 1 Produced by Disruption of Cysteine Bridges

**DOI:** 10.3390/nu9020171

**Published:** 2017-02-21

**Authors:** Pathum Dhanapala, Dulashi Withanage-Dona, Mimi L. K. Tang, Tim Doran, Cenk Suphioglu

**Affiliations:** 1 Neuro Allergy Research Laboratory (NARL), School of Life and Environmental Sciences, Faculty of Science, Engineering and Built Environment, Deakin University, 75 Pigdons Road, Geelong 3216 VIC, Australia; dpathum@deakin.edu.au or pdhanapala@bwh.harvard.edu (P.D.); awitha@deakin.edu.au (D.W.-D.); 2 Australian Animal Health Laboratory (AAHL), Biosecurity Flagship, Commonwealth Scientific and Industrial Research Organisation (CSIRO), 5 Portarlington Road, East Geelong 3219 VIC, Australia; Timothy.Doran@csiro.au; 3 Poultry CRC, P.O. Box U242, University of New England, Armidale 2351 NSW, Australia; 4 Department of Orthopedic Surgery, Brigham and Women’s Hospital, Harvard Medical School, 60 Fenwood Road, Boston, 02115 MA, USA; 5 Department of Allergy and Immunology, Royal Children’s Hospital, 50 Flemington Road, Parkville 3052 VIC, Australia; Mimi.Tang@rch.org.au; 6 Allergy and Immune Disorders, Murdoch Children’s Research Institute, 50 Flemington Road, Parkville 3052 VIC, Australia; 7 The University of Melbourne, Parkville 3010 VIC, Australia

**Keywords:** allergens, egg allergy, immunotherapy, hypoallergens

## Abstract

Background: Gal d 1 (ovomucoid) is the dominant allergen in the chicken egg white. Hypoallergenic variants of this allergen can be used in immunotherapy as an egg allergy treatment approach. We hypothesised that disruption of two of the nine cysteine-cysteine bridges by site-directed mutagenesis will allow the production of a hypoallergenic variant of the protein; Methods: Two cysteine residues at C192 and C210 in domain III of the protein were mutated to alanine using site-directed mutagenesis, to disrupt two separate cysteine-cysteine bridges. The mutated and non-mutated proteins were expressed in *Escherichia coli* (*E. coli*) by induction with isopropyl β-d-1-thiogalactopyranoside (IPTG). The expressed proteins were analysed using sodium dodecyl sulfate polyacrylamide gel electrophoresis (SDS-PAGE) and immunoblotting to confirm expression. Immunoglobulin E (IgE) reactivity of the two proteins was analysed, by immunoblotting, against a pool of egg-allergic patients’ sera. A pool of non-allergic patients’ sera was also used in a separate blot as a negative control; Results: Mutant Gal d 1 showed diminished IgE reactivity in the immunoblot by showing lighter bands when compared to the non-mutated version, although there was more of the mutant protein immobilised on the membrane when compared to the wild-type protein. The non-allergic negative control showed no bands, indicating an absence of non-specific binding of secondary antibody to the proteins; Conclusion: Disruption of two cysteine bridges in domain III of Gal d 1 reduces IgE reactivity. Following downstream laboratory and clinical testing, this mutant protein can be used in immunotherapy to induce tolerance to Gal d 1 and in egg allergy diagnosis.

## 1. Introduction

Hypersensitivity to chicken egg is caused by allergens present in the egg white and egg yolk. Among these, Gal d 1 (ovomucoid) is known to be the most allergenic and predominant allergen and it is found in the chicken egg white [1,2]. This 28 kDa glycoprotein accounts for approximately 11% of the total egg white protein. The tertiary structure of Gal d 1 is composed of 186 amino acids which form three domains, with each domain containing approximately 60 amino acids. The tertiary structure is robustly supported by nine intra-domain cysteine-cysteine disulphide bridges and five oligosaccharide side chains. The function of Gal d 1 is known to be a trypsin inhibitor; however, the trypsin inhibitory activity is limited to the second domain [1]. Hypersensitivity to Gal d 1 occurs because of its ability to efficiently bind to immunoglobulin E (IgE). It has eight IgE binding epitopes [2], some of which are linear while others are conformational. The highly IgE-reactive epitopes present in the third domain make it the most allergenic domain of the three. The presence of linear IgE binding epitopes in Gal d 1 makes it resistant to conditions such as heat and/or proteolytic digestion [3]. Since egg-allergic patients are often allergic to cooked egg [4], it can be suggested that Gal d 1 plays a crucial role in cooked egg allergy due to its rigidity. These specific features of Gal d 1 make it the prime allergen when compared to other allergens in chicken egg and an ideal target for the development of egg allergy treatment strategies.

There is no long-term cure for egg allergy. Strict avoidance of egg is the currently recommended management strategy; however, avoidance is difficult and may cause malnutrition in children [5,6], especially in financially disadvantaged families where procurement of more expensive nutritional supplements or food that can replace eggs may be difficult. It is also problematic to completely avoid eggs because of the presence of components or traces of egg in various food products, pharmaceutical products and vaccines [7,8]. Allergen-specific oral immunotherapy (OIT) offers a potential treatment strategy, not only for egg allergy but also for other types of food allergies. OIT essentially involves the gradual oral feeding of an allergen to the patient in order to induce tolerance [9,10]. However, OIT can be perilous for some patients, primarily because of the high allergenecity of some allergens and the sensitivity of the patient, which may cause adverse conditions such as anaphylaxis that can even lead to death [11,12,13]. Adverse reactions to OIT are currently a potential barrier to clinical application [14,15]. Therefore, production of less allergenic versions, or hypoallergens, of allergens has been the focus of many research groups [16,17,18], because these hypoallergens can offer improved safety of oral immunotherapy. 

Production of hypoallergenic Gal d 1 can be achieved by using mutagenesis as a tool in two different strategies: the first is by mutating the sequences of the IgE binding epitopes and the second is by targeting the secondary structure of the proteins. Drew et al. (2004) [19] successfully produced a hypoallergenic variant of the major latex allergen Hev b 6.10 by disrupting the cysteine-cysteine bonds of the protein to reduce its IgE reactivity. In this study, we have successfully produced a hypoallergenic variant of Gal d 1 by targeting only two of the nine cysteine-cysteine bridges using site-directed mutagenesis.

## 2. Methods

### 2.1. Site-Directed Mutagenesis of Gal d 1

**Figure 1:**
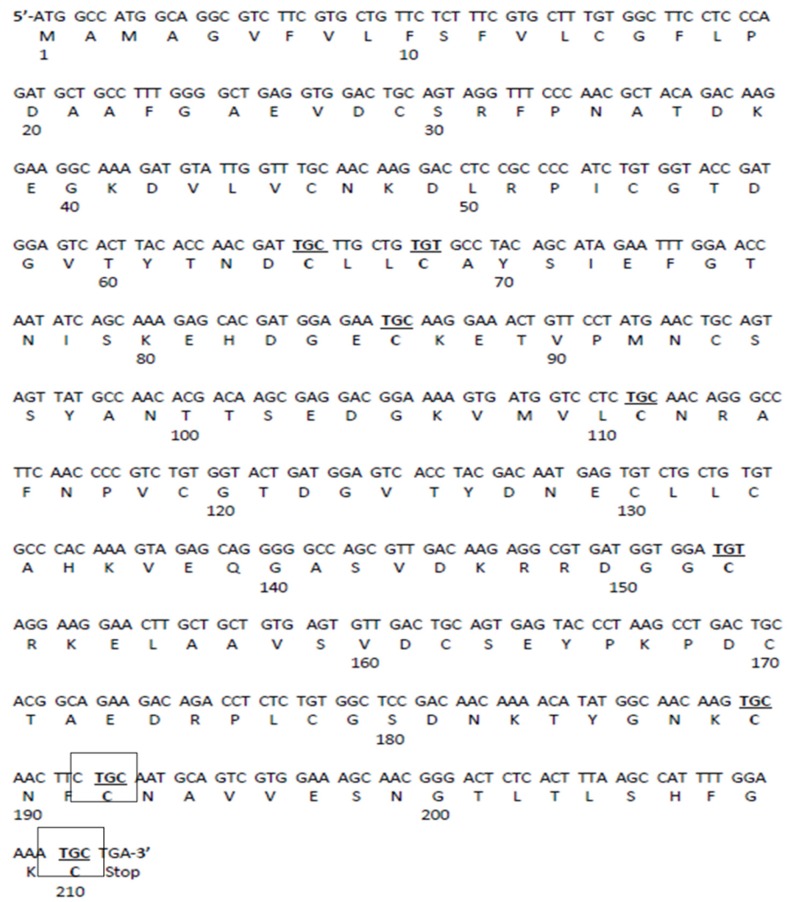
The nucleotide and amino acid sequence of Gal d 1. The squared cysteine (C) residues at positions C192 and C210 are the targeted residues. These were replaced with alanine by mutating the nucleotides to GCC.

**Figure 2:**
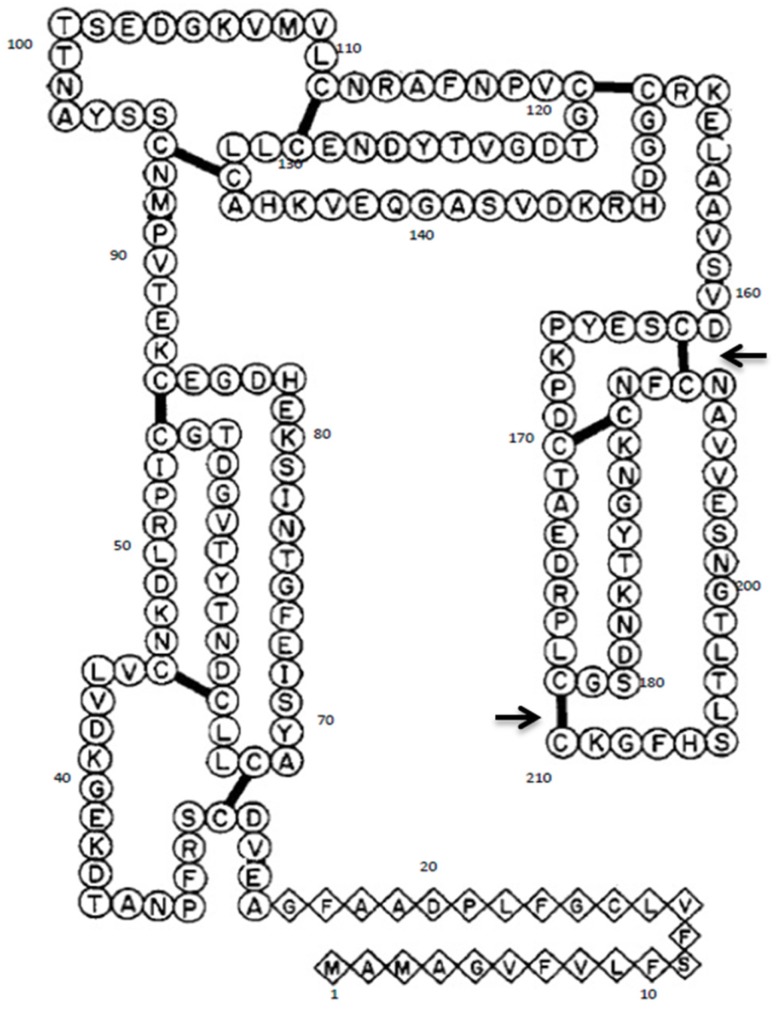
The secondary structure of Gal d 1 showing the total number of cysteine bridges. The two arrows show the two cysteine bridges that would be destroyed by the mutations shown in Figure 1. Figure adapted from: Kato et al., 1987 [1].

The cDNA of Gal d 1 was cloned into pTrcHisA expression vector as discussed in Dhanapala et al. 2015 [20]. This construct was used for site-directed mutagenesis of nucleotides coding two cysteine residues, using QuickChange Lightning Multi Site-Directed Mutagenesis kit (Agilent Technologies, Santa Clara, CA, USA). Two TGC triplicates coding for cysteine 192 and 210 (Figure 1) were targeted in order to disrupt two different cysteine-cysteine bridges located in domain III of Gal d 1 (Figure 2). The TGC codons were changed to GCC codons that code for alanine. Initially, mutagenic primer pairs were designed according to the mutagenesis kit guidelines. The two pairs were named PM7 and PM9, because the mutations were targeting the seventh and the ninth cysteine-cysteine bridges, respectively. The primers are as follows; PM7 forward 5′-GGCAACAAGTGCAACTTC**GCC**AATG CAGTCGTGGAAAG-3′, PM7 reverse 5′-CTTTCCACGACTGCATTGGCGAAGTTGCACTTGTTGCC-3′, PM9 forward 5′-ACTCTCACTTTAAGCCATTTTGGAAAA**GCC**TGAAAGCTTGGCTGT-3′, PM9 reverse 5′-ACAGCCAAGCTTTCAGGCTTTTCCAAAATGGCTTAAAGTGAGAGT-3′. The bolded and underlined GCC on forward primers show the mutations. To mutate the Gal d 1 cDNA in pTrcHisA vector, the above mentioned primers and the cDNA constructs (as template DNA) were subjected to a polymerase chain reaction (PCR). The PCR reaction was set up according to Table 1. The PCR was then run according to the cycling parameters outlined in Table 2. Following the PCR, the reaction was digested with *Dpn* I for 5 min at 37 °C, to digest the non-mutated template DNA.

### 2.2. Chemical Transformation into E. coli

The mutated plasmids were then transformed into XL10-Gold ultracompetent *E. coli* cells following manufacturer’s guidelines provided with the mutagenesis kit. The reaction was incubated with 0.5 mL of pre-heated Luria broth (LB) media at 37 °C for 1 h at 250 rpm. The transformant was then spread-plated on LB agar with 50 µg/mL ampicillin and incubated overnight at 37 °C. The next day, 6 clones were grown in fresh LB media with ampicillin and grown overnight. The cells in overnight cultures were pelleted by centrifuging at 13,000 rpm for 5 min and subjected to a mini-prep (Qiagen, Hildon, Germany) to isolate the plasmid constructs following manufacturer’s guidelines. The isolated plasmids of the six clones were sequenced to confirm the mutations. The sequences were aligned and compared with wild-type Gal d 1 using the NCBI BLAST tool. The clones that had the correct sequence and the mutations were then transformed into *Express I^q^* chemically competent *E. coli* cells (New England BioLabs, Boston, MA, USA) following manufacturer’s guidelines. The transformants were plated on LB agar with ampicillin and incubated overnight at 37 °C. In addition to the mutant transformants plate, a sample of glycerol-stocked *E. coli* containing the wild-type ovoumucoid construct was also plated on LB agar with ampicillin. 

### 2.3. Time-Course Expression of Mutant Gal d 1 to Determine Optimum Expression Time

A single colony of the mutant Gal d 1 was grown overnight in LB media with 50 µg/mL ampicillin. The overnight culture was then subcultured in 10 mL of fresh LB media and grown to mid-log phase (OD_600_ 0.4–0.6). A 1 mL sample of the cells was pelleted to be used as the unexpressed control (0 h) of the time-course expression. Expression was then induced with 40 µL of IPTG and the cells were incubated for 6 h at 37 °C with shaking at 250 rpm. A 1 mL sample was collected every one hour for the 6 h period. The pellets collected at time points 0, 2, 4, 5 and 6 were lysed using 400 µL of Cell Lytic B (Sigma Aldrich, Natick, MA, USA) lysis reagent and centrifuged at 13,000× *g* for 5 min to separate the pellet (insoluble fraction) and the supernatant (soluble fraction). The two fractions were analysed using SDS-PAGE and western blot according to the methods described in Dhanapala et al. 2015 [20]. 

### 2.4. Expression and Immunoblotting of Wild-Type and Mutant Gal d 1 Using Three Different Detection Antibodies

The wild-type and mutant Gal d 1 were expressed in *E. coli* to their optimum time points as determined by the time-course expressions (wild-type Gal d 1 optimum time was determined in Dhanapala et al. 2015 [20]). Cells were pelleted and lysed using Cell Lytic B as previously described. The soluble fractions of both proteins were run on SDS-PAGE in equal amounts (15 µL), along with a molecular weight marker. A gap lane was left between the two proteins to avoid any cross-contamination between the two variants. The SDS gel was then transferred on to a nitrocellulose membrane to be used for western blotting. A total of five nitrocellulose membranes were prepared this way, of which two would be used in the analysis described in Section 2.5. Three prepared nitrocellulose membranes were subjected to Western blotting using three different antibodies that can detect the expressed protein (e.g., anti-Xpress antibody, tetra-His antibody and penta-His antibody). 

### 2.5. Immunological Analysis of Wild-Type vs. Mutant Gal d 1 Using Western Blot

The two remaining nitrocellulose membranes from Section 2.4 were used for immunoblotting using egg allergic and non-allergic patients’ sera to test for IgE reactivity. In a previous study, we used a pool of egg allergic patients’ sera and a pool of non-allergic patients’ sera for immunological analysis of recombinant egg white proteins [20]. In this study we used the same pooled serum preparations and incubated one membrane with allergic patients’ sera and the other with non-allergic patient’s sera, and incubated overnight at 4 °C. The blots were then incubated with anti-human IgE (alkaline phosphatase conjugated) secondary antibody produced in goat at a dilution of 1:1000. The bands were detected using a chromogenic substrate as used in the Western blots described in Section 2.4. 

## 3. Results

### 3.1. Mutagenesis of Gal d 1

Following site-directed mutagenesis to alter C192 and C210, six clones were sequenced to confirm the mutations. Five of the six clones had only one mutation present. One clone had both of the mutations at the expected locations of the sequence. When the wild-type Gal d 1 sequence was aligned with the mutant Gal d 1 sequence on NCBI BLAST, it was seen that the TGC codons (cysteine) for C192 and C210 had been changed to GCC, which in turn codes for alanine.

### 3.2. Time-Course Expression of Mutant Gal d 1 to Determine Optimum Expression Time

**Figure 3:**
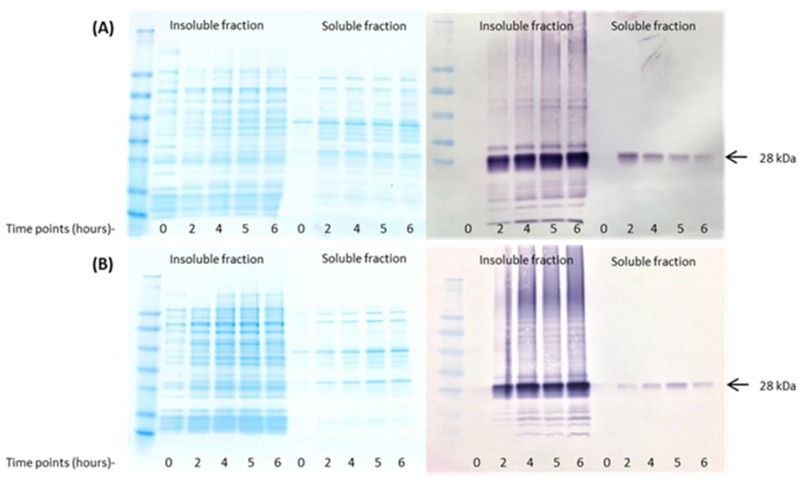
Time-course expression of the mutant Gal d 1. A time-course expression of the wild-type Gal d 1 (**A**) was previously published in Dhanapala et al. 2015 [20]. The mutant Gal d 1 (**B**) was subjected to a time-course expression to determine its optimal expression time and conditions and was compared to the wild-type Gal d 1 expression shown in (**A**).

The mutant Gal d 1 protein was expressed in *E. coli* following IPTG induction for 6 h, and pellets were collected every 1 h, including one before IPTG induction. The pellets from time points 0, 2, 4, 5 and 6 were lysed and the soluble and insoluble fractions were analysed using SDS-PAGE and Western blot. The results show that the optimum expression time point for mutant Gal d 1 is 5 h (Figure 3B), as compared to 2 h for wild-type Gal d 1 (Figure 3A) [20]. It can also be seen that the expression level of mutant Gal d 1 decreased after 5 h. 

### 3.3. Expression and Immunoblotting of Wild-Type and Mutant Gal d 1 Using Three Different Detection Antibodies

**Figure 4:**
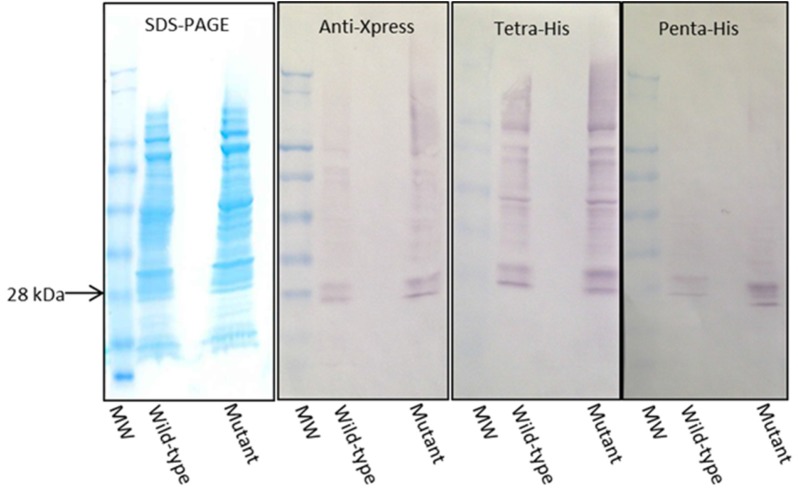
Immunoblot comparison of the wild-type and mutant Gal d 1 immobilised on nitrocellulose. Three Western blots were conducted using His-tag–specific antibodies (Tetra-His & Penta-His) and anti-Xpress antibody to compare the expression level of wild-type and mutant (PM7/9) Gal d 1. SDS-PAGE shows the profile of the loaded proteins.

The wild-type and mutant recombinant Gal d 1 proteins were expressed in LB until their respective optimum time points by induction with IPTG. The proteins were analysed by SDS-PAGE and Western blotting using three different antibodies (anti-Xpress, Tetra-His and Penta-His antibodies). The SDS-PAGE shows that similar amounts of both proteins were loaded on to the gel (Figure 4). The Western blots show that there was a slightly higher amount of mutant protein present on the nitrocellulose membrane (Figure 4).

### 3.4. Immunological Analysis of Wild-Type vs. Mutant Gal d 1 Using Western Blot

**Figure 5:**
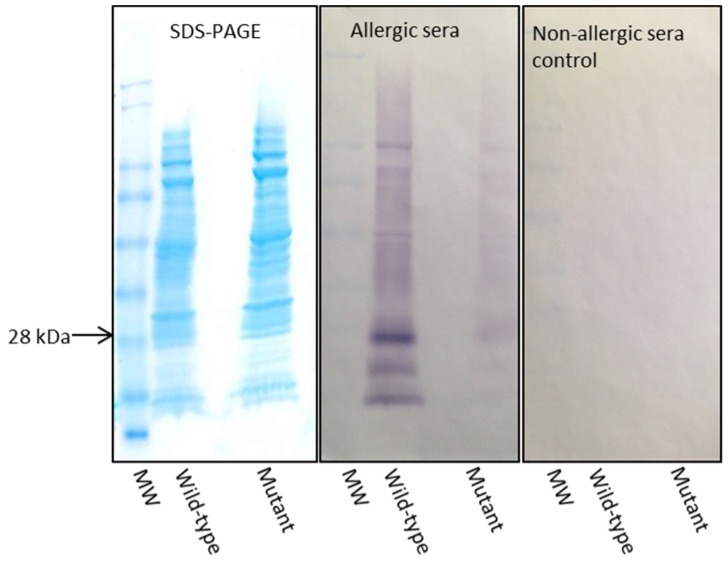
Immunological comparison of IgE reactivity of wild-type and mutant Gal d 1. Western blots were conducted, with exactly the same amount of proteins loaded against egg-allergic and non-allergic patients’ sera. Anti-human IgE produced in goat was used as the secondary antibody. Non-allergic controls were used to test for any non-specific binding of secondary antibody. The blots show a loss of IgE reactivity in the mutant PM7/9.

Two nitrocellulose membranes were prepared using the same samples used for the blots shown in Figure 4. The two membranes were subjected to Western blotting using egg-allergic patients’ sera and non-allergic sera. The egg-allergic patients’ sera blot showed reduced binding (lighter colouration) for the mutant Gal d 1 lane when compared to the wild-type Gal d 1 (Figure 5). The non-allergic sera blot showed no detectable bands in either of the lanes representing wild-type or mutant Gal d 1 (Figure 5). 

## 4. Discussion

Hypersensitivity to chicken egg white is mainly caused by four major egg white allergens. Of these, Gal d 1 is known to be the most allergenic protein. Gal d 1 is known to cause hypersensitivity in its natural or cooked form. This may primarily be due to its rigid tertiary structure which allows it to withstand harsh conditions such as heat and stomach/digestive acids. Due to the lack of an effective curative treatment, strict avoidance is currently the standard method of managing egg allergy. However, this strategy is not feasible due to the difficulty in achieving complete egg avoidance and the high nutritional value of eggs in a balanced diet, especially for children. Induction of tolerance to allergens is a well-established strategy for treatment of different types of allergies such as insect venom or pollen allergy. Immunotherapy, specifically oral immunotherapy (OIT), which is a type of allergen-specific immunotherapy (SIT), has been explored for the induction of tolerance to food allergens. OIT involves feeding a patient increasing amounts of raw or cooked versions of the allergen source, in order to induce desensitization or long-lasting tolerance to the allergen [21]. One barrier to implementation of OIT in the clinical setting is the high rate of adverse reactions necessitating discontinuation of therapy, which primarily involve immediate allergic reactions to the allergen [14,15]. Recombinant versions of allergens offer an approach to reduce adverse reactions, thereby allowing improved effectiveness [22]. These recombinant allergens are purer and free from contamination from other allergens of the food source, and thus may also be useful for the diagnosis of allergy (e.g., skin prick tests or immunoassay).

Food allergies, including allergy to chicken egg, may sometimes cause severe reactions such as anaphylaxis. In such patients, use of natural allergens for diagnosis or immunotherapy may be associated with unwanted allergic reactions. Therefore hypoallergenic, or less allergenic, versions of allergens would be useful in such patients with severe allergic reactions. Production of hypoallergenic variants has been rigorously pursued in allergy research, for example the production of a hypoallergenic variant of the major latex allergen Hev b 6.01 by site-directed mutagenesis by Drew et al., 2004 [19], and the development of a vaccine using hypoallergenic derivatives of the birch pollen allergen Bet V 1 by Niederberger et al., 2004 [23]. In this study, we developed a hypoallergenic variant of the major egg white allergen Gal d 1 (Gal d 1) which showed reduced IgE reactivity when compared to its wild-type counterpart. 

For mutagenesis, it was decided to use alanine as a replacement for cysteine residues at C192 and C210 because it is the most common amino acid that does not have extreme electrostatic or steric effects on the conformation of the protein [24]. The sequencing result of the six clones post-mutagenesis showed that five clones had only one of the desired mutations present. The mutagenesis kit used in this study allowed introducing multiple mutations in a single reaction. Therefore, the low efficiency can be attributed to factors such as the quality of the template DNA or the efficiency of the mutagenic primers. Nevertheless, one clone had both of the desired mutations at C192 and C210, replacing TGC codons (cysteine) with GCC (alanine). The Gal d 1 secondary structure is made up of three tandem domains (I–III), with domain III showing high IgE reactivity [25]. By targeting C192 and C210, we aimed to destroy two cysteine-cysteine disulphide bridges in domain III, thus altering its conformation. We hypothesised that altering the conformation of domain III may have a significant effect on IgE reactivity of the whole protein. 

The mutant Gal d 1 was successfully expressed in *E. coli*. A time-course expression was conducted to determine the optimum time point for the expression of the mutant protein. We previously reported that the wild-type recombinant Gal d 1 was best expressed at 2 h post-induction with IPTG [20]. However, the expression pattern of the mutant protein was different to that of the wild-type, as shown in Figure 3. The mutant protein’s expression level increased with time up until 5 h, as opposed to the wild-type protein’s which showed a reduction in expression after 2 h. Similar to the wild-type, the mutant was highly expressed in the insoluble fraction, indicating that the expression of the protein causes the formation of inclusion bodies in *E. coli*. Nonetheless, the amount expressed in the soluble fraction was sufficient for the remainder of this study. 

When analysing two proteins on an immunoblot to compare their reactivity for an antibody, it is essential to immobilise similar amounts of the two proteins. When comparing recombinant proteins, it is crucial that the proteins are purified to allow loading of similar amounts of proteins to a gel to be transferred on to a nitrocellulose membrane. In this study, we did not have purified recombinant versions of the wild-type or mutant Gal d 1. Therefore, after inducing expression until the optimum time point of each variant, we loaded similar volumes of the crude *E. coli* extracts onto gels, transferred on to nitrocellulose and subjected to detection using different antibodies to confirm that both proteins are expressed and loaded at similar quantities. When analysed on SDS-PAGE and Western blotted using Anti-Xpress, Tetra-His and Penta-His antibodies, it was evident that there was more of the mutant protein immobilised on the nitrocellulose membrane when compared to the wild-type protein. This was not a significant issue as we were testing the IgE reactivity of the mutant against the wild-type Gal d 1. It was only vital to ensure that the wild-type protein did not exceed the amount of mutant protein on the membrane. Following the aforementioned immunoassays, the two proteins were compared against each other for IgE reactivity using egg-allergic patients’ sera. The blot in Figure 5 clearly shows that there is a significantly visible reduction of IgE reactivity in the mutant protein, although there is more of the mutant protein immobilised on the membrane. One may argue that the IgE in the sera may have attached/reacted to *E. coli* protein; however, we have previously shown that the IgE in the egg-allergic sera we used did not react to *E. coli* proteins [20]. The membrane incubated with non-allergic sera showed no bands, indicating that the secondary antibody, anti-human IgE produced in goat, does not non-specifically bind to the recombinant proteins. Furthermore, breaking down and accumulation of the protein was evident by the presence of multiple bands on the immunoblots on Figure 4. The presence of less bands in the Penta-His antibody blot, compared to Tetra-His, can be attributed to the detection of proteins broken down at the histidine tag by the Tetra-His antibody. 

This study shows that disruption of only two out of nine cysteine-cysteine bridges in Gal d 1 by targeting C192 and C210 significantly reduces its reactivity to egg-specific IgE. The result also suggests that the structure of the protein plays a crucial role in its allergenecity. This mutant Gal d 1 has the potential to be used in safer egg oral immunotherapy. This study provides preliminary results for future research involving the production of hypoallergenic variants of egg allergens, in particular Gal d 1. The result obtained from this study should be followed by further in vitro and in vivo experimentation. The foremost next step is purification of the protein from the soluble fraction of *E. coli*. We have expressed the protein with a 6× histidine tag; therefore, nickel affinity purification techniques can be utilsed for this purpose. The purified protein can then be used in B-cell and T-cell activation tests/assays. T-lymphocytes (T-cells) are known to be important in allergic desensitization [26,27]; therefore, it is imperative to test the ability of the hypoallergenic Gal d 1 produced in this study to stimulate T-cells. Animal models also play a pivotal role in food allergen research [28], and therefore it should be suggested that the hypoallergenic Gal d 1 we produced should undergo animal model–based experimentation prior to clinical testing. We have previously shown that Gal d 1 is more reactive in comparison to other allergens when tested against egg-allergic patients’ sera [20]. In addition, the same study showed that patients show reactivity to more than one allergen, even the patients showing high reactivity to Gal d 1. Therefore, it should be highlighted that a hypoallergenic variant of Gal d 1 is only useful for reducing allergic response in patients allergic to multiple allergens during immunotherapy, rather than complete abolition of reactivity, thus showing the importance of research into the development of hypoallergenic variants of other allergens in the egg. 

## 5. Conclusions

In summary, we have successfully produced a hypoallergenic variant of the major egg white allergen Gal d 1 by disrupting two cysteine-cysteine bridges using site-directed mutagenesis. This hypoallergenic variant, upon purification and further immunological analysis, may be used as an excellent constituent in future immunotherapy vaccines for egg allergy.

## Figures and Tables

**Table 1:** Mutagenic polymerase chain reaction (PCR) master mix components.

Reaction Component	Volume Used (µL)
10× QuickChange Lightning Multi reaction buffer	2.5
Double-distilled water	15.5
Template DNA	1 (50 ng)
Mutagenic primers	1 of each primer (100 ng of each primer)
Deoxy-nucleoside triphosphate (dNTP) mix	1
QuickChange Lightning Multi enzyme blend	1
Total	25

**Table 2:** Mutagenic PCR conditions.

Segment	Cycles	Temperature	Time
1	1	95 °C	2 min
2	30	95 °C	20 s
		55 °C	30 s
		65 °C	3 min (30 s/kb of plasmid length)
3	1	65 °C	5

## Abbreviations

IgE	immunoglobulin E
OIT	oral immunotherapy
SPT	skin prick tests
